# Editorial: Advances in the biological effects of ionizing radiation

**DOI:** 10.3389/fonc.2023.1352771

**Published:** 2024-01-03

**Authors:** Fada Guan, Lina Zhao, Julianna K. Bronk, Mirjana Maletic-Savatic, David R. Grosshans, David J. Carlson

**Affiliations:** ^1^ Department of Therapeutic Radiology, Yale University School of Medicine, New Haven, CT, United States; ^2^ Department of Radiation Oncology, Xijing Hospital, Xi’an, China; ^3^ Department of Radiation Oncology, MD Anderson Cancer Center, Houston, TX, United States; ^4^ Department of Pediatrics, Division of Child Neurology, Baylor College of Medicine, Houston, TX, United States

**Keywords:** radiation biology, abscopal effect of radiation therapy, radiosensitivity, radioresistance, radiomics

Ionizing radiation is a double-edged sword. If applied appropriately, it can destroy the malignancy and cure cancer patients; however, it can also cause temporary or permanent adverse effects to normal tissues and organs. With advancements in technology and medicine that allow for higher rates of disease cure, many cancer survivors must live with life-long side effects from their cancer therapies, including toxicities from radiation. In rare instances, serious therapy-related side effects can even lead to patient death. Therefore, continued investigation toward minimizing radiation-induced toxicities while maintaining tumor control is a current and paramount unmet clinical need.

Radiation therapy for cancer treatment is grounded in the basic principle of maximizing tumor cell kill while simultaneously sparing normal tissues. Notably, radiosensitivity of tumors and normal tissues are intrinsically heterogenous, wherein some regions are radioresistant while others may be radiosensitive. Radiosensitivity also depends on a variety of factors such as tissue oxygen concentration and radiation beam quality. Inter- and intra-tissue radiosensitivity have increased the complexity and workload in designing and implementing effective treatment plans for many disease sites. Even for the same disease, clinical outcomes can vary among patients due to individual differences in response to radiation.

Although our understanding of the underlying mechanisms of radiation-induced biological effects has greatly improved over years, there remains many unknowns. The aim of the present Research Topic is to provide an up-to-date overview of innovations and recent developments in experimental techniques, computational methods, biological effects modeling, and novel data analysis tools in radiobiology studies. The goal of this Research Topic is to collect and summarize the growing knowledge in the advancement of biological effects of ionizing radiation, particularly in radiation oncology for cancer treatment.

Four manuscripts are published in the current Research Topic. The first manuscript is a case report by Cerbon et al. presenting the abscopal effect observed in visceral and osseous metastases after liver stereotactic body radiation therapy (SBRT). The authors state that this is the first case that describes the complete abscopal response in a sinonasal mucosal melanoma histology and the first report of abscopal response following liver SBRT in combination with nivolumab and relatlimab for metastatic sinonasal mucosal melanoma in the setting of both visceral and osseous lesions. These findings suggest that the combination of SBRT with first-line immunotherapy potentiates the adaptive immune response and is a viable path for immune-mediated tumor rejection. Nevertheless, the work presented in this case report is acknowledged to be hypothesis-generating and the authors propose that further investigation of the mechanisms behind this response could be an active Research Topic with exceedingly promising potential.

The second manuscript is a review on the possible mechanisms of radiotherapy resistance in cervical cancer by Zhang et al. The authors systematically summarize the current reported radiotherapy-resistance mechanisms of cervical cancer in an analysis of approximately 200 publications. Most findings support that radiation resistance is associated with a reduction in cervical cancer cell death due to enhanced DNA damage repair ability after radiotherapy. Another important mechanism is related to cancer stem cells radio-resistance secondary to improved DNA repair capacity. Additional radiotherapy resistance mechanisms in cervical cancer discussed in this analysis include hypoxia, tumor microenvironment, glucose, amino acid, lipid metabolism, cell cycle and apoptosis, and microRNAs and lncRNAs. Notably, multiple mechanisms may play effective roles together in radiation resistance. For example, hypoxia, glycolysis, and cancer stem cells could exist together in the tumor microenvironment.

The third manuscript is an original research article by Tobiasz et al. The authors applied a dynamic programming algorithm to create a multivariate piecewise linear regression model to predict radiosensitivity using the association with the genome-wide copy number variation. They used the Affymetrix CytoScan HD microarrays to survey common copy number variations as markers of radiation sensitivity in 129 fibroblast cell strains. Radiosensitivity was measured by the surviving fraction of cells at 2 Gy. The authors concluded that implementation of the multivariate linear regression method could help narrow the set of copy number variation makers from the whole radiosensitivity range to the smaller intervals of interest. The significance of this work is that segmented-related markers can be used to identify radiotherapy patients who are most radiosensitive and require reduced doses to avoid complications and the most radioresistant eligible for dose escalation to improve outcomes.

The fourth manuscript is an original research article by Montoya et al. that aimed at predicting the treatment response to immunotherapy in advanced non-small cell lung cancer (aNSCLC) patients using a murine model and radiomics data of both patients and mice. Although immunotherapy improves the disease response of aNSCLC compared with conventional chemotherapy, resistance to immunotherapy remains common. The significance of this work lies in the fact that predicting responses could help guide selection of intensified or alternative anti-cancer regimens. Pretreatment radiomics features were used in this retrospective study to develop models predictive of immunotherapy response both in mice and in patients. Identical radiomics features and neutrophil-to-lymphocyte ratio correlated with outcomes and immune checkpoint inhibitor response in two independent patient cohorts supporting the generalizability and translatability of the author’s findings.

With a broad spectrum of topics included in this Research Topic on the advancement of the biological effects of ionizing radiation, this Research Topic highlights the implications of recent research findings and systematic review in multiple domains. These include treatment response prediction using preclinical data-based models, proposed mechanisms of radiotherapy resistance, and characterization of the abscopal effect of radiation therapy. It is our hope that these findings may advance efforts toward clinical implementation of personalized cancer patient treatment, ultimately, to improve clinical outcomes and reduce complications.

## Author contributions

FG: Writing – original draft. LZ: Writing – original draft. JB: Writing – review & editing. MM-S: Writing – review & editing. DG: Writing – review & editing. DC: Writing – review & editing.

